# When to start antiretroviral treatment? A history and analysis of a scientific controversy

**DOI:** 10.4102/sajhivmed.v18i1.734

**Published:** 2017-12-05

**Authors:** Nathan Geffen, Marcus O. Low

**Affiliations:** 1 Department of Computer Science and Centre for Social Science Research, University of Cape Town, South Africa; 2 Spotlight Magazine, Cape Town, South Africa

## Abstract

**Background:**

Since 1987 HIV scientists and activists have debated the optimal point to start antiretroviral treatment. Positions have varied between treating people with HIV as soon as they are diagnosed, based on biological, modelling and observational evidence, versus delaying treatment until points in disease progression at which clinical trial evidence has shown unequivocally that treatment is beneficial.

**Objectives:**

Examining the conduct and resolution of this debate may provide insight into how science works in practice. It also documents an important part of the history of the HIV epidemic.

**Method:**

We describe clinical trials, observational studies, models and various documents that have advanced the debate from 1987 to 2015.

**Results and conclusion:**

Evidence accumulated over the past decade, especially from randomised controlled clinical trials, has shown that immediate treatment both reduces the mortality and the risk of HIV transmission; it benefits both public health and the individual patient. By mid-2015, the debate was resolved in favour of immediate treatment.

## Introduction

Since the publication of the first antiretroviral trial in 1987, scientists and patient advocates have debated the optimal time for people with HIV to start antiretroviral treatment. Guidelines, both national and international, have changed back and forth on this question, reflecting changes in expert opinion and new scientific developments.

We describe the when-to-start debate and its resolution in mid-2015. This debate exemplifies the problem of deciding policy when the evidence is still being collected, or how ‘technological decision making’ is done when there is ‘scientific uncertainty’.^1^ While much has been written about the debate over the cause of AIDS, a consequence mainly of former South African President Thabo Mbeki’s views, little has been written on the when-to-start debate. Yet, there is more to be learnt about how science works from the when-to-start debate. This is because it was genuinely hard to determine public health policy from the limited evidence. By contrast, the science that HIV is the cause of AIDS was clear, and that debate was fuelled not by legitimate scientific disagreements, but by politics and ideology.

The question of when-to-start treatment was contested not only between scientists, but also between AIDS activists. Participants with reasonable claims to expertise who for the most part were familiar with the same scientific literature reached opposing conclusions on what treatment guidelines should recommend.

The participants in the debate differed in their assessments of the value of observational versus clinical trial data. They also differed on whether the public health benefits of reducing HIV transmission by treating people earlier outweighed the unknown harms to individual patients because of side effects of drugs, difficulties with adherence to lifelong medication and the development of drug resistance. And they differed on how much value to assign mathematical models and observational data. The stakes were high: the contestants understood that settling the question of when-to-start treatment might have considerable effects on life expectancy and the incidence of HIV.

If we think about the when-to-start debate as a court case, then the main exhibits were a mathematical model by Granich et al.^2^ which showed that a policy of universal testing followed by immediate treatment of people with HIV would lead to the eradication of the disease; a clinical trial that showed that people with HIV on antiretroviral treatment are unlikely to transmit the virus^3^; several observational studies, with inconsistent results, which compared what happened to patients who started treatment at different stages of HIV infection; and a massive multinational clinical trial called Strategic Timing of Antiretroviral Treatment (START).^4^ Besides these exhibits, there were many others that either supported or contested some of the main ones.

The publication of results of the START trial in August 2015, a year-and-a-half ahead of schedule, effectively resolved the question of when-to-start treatment, generating broad scientific consensus on the question. But it did not and could not resolve differences in values and methodologies of the debate’s participants. These differing values and methodologies in the approach to resolving medical science questions will continue, perhaps indefinitely, to be the subject of sociological and philosophical enquiry.

## Background

The results of the first randomised controlled antiretroviral clinical trial, BW002, were published in 1987.^5^ For 24 weeks, people with AIDS received azidothymidine (now better known as AZT or zidovudine) or placebo. Of the 145 participants who received AZT, one died, compared to 19 out of 137 who received placebo.

Despite this promising result, the trial was too short to show that monotherapy soon results in drug resistance followed by most patients developing AIDS illnesses again. New combination treatments were needed to reduce the risk of resistance.

New antiretrovirals went to trial and were approved by the United States Food and Drug Administration (FDA) through the 1990s: didanosine (1991), zalcitabine (1992), stavudine (1994) and lamivudine (1995). It was, however, the development of protease inhibitors and non-nucleoside reverse transcriptase inhibitors – such as saquinavir (1995), ritonavir (1996), indinavir (1996) and nevirapine (1996) – which changed the nature of HIV treatment.^6^ Arts and Hazuda^6^ write, ‘The advent of combination therapy, also known as HAART, for the treatment of HIV-1 infection was seminal in reducing the morbidity and mortality associated with HIV-1 infection and AIDS’. People with HIV on combination therapy, typically three antiretrovirals taken daily for life, who adhere to their regimen have a very small risk of resistance. The virus can remain suppressed indefinitely restoring near-normal life expectancy.^7^

Today there are about 25 individual antiretroviral drugs spread over six different classes (i.e. differing modes of action) approved by the FDA.^8^ But it was only in the second half of 2015, 28 years after the completion of the first randomised controlled antiretroviral trial that the answer to the when-to-start question was settled.

## Changing guidelines

The two main criteria in treatment guidelines for determining when to start treatment have been symptoms of AIDS and CD4 T-lymphocyte count. The ‘to and fro’ of treatment guideline changes has previously been described.^9^ When AZT was approved in 1987, the US Department for Health and Human Services (DHHS) set the CD4 threshold at 500 cells/µL. In April 2001, it was reduced to 350, and then to 200 in 2003. In 2007, it was raised to 350, and then 500 in 2009. In 2013, CD4 count was removed as a criterion for determining when-to-start treatment. In 2003, the World Health Organization (WHO) guidelines – produced for resource-limited settings – set the CD4 threshold at 200 cells/µL. This increased to 350 in 2010 and then 500 in 2013, with a recommendation that some groups of patients start irrespective of CD4 count. Changes over time in the CD4 initiation threshold can be found in the South African Department of Health’s, British HIV Association’s and European AIDS Clinical Society’s guidelines. And often, they were not in sync with each other. For example, in 2012, these differed from the DHHS guidelines by retaining the 350 threshold. South Africa’s guidelines have changed from 200 cells/µL to 350 to 500, followed by treatment irrespective of CD4 count.

One of the reasons why many scientists, clinicians and activists in the late 1990s and the early 2000s were reluctant to endorse early treatment for people was the surprising results of the Concorde trial.^10^ Symptom-free people with HIV were enrolled in the trial from 1988 to 1991. Follow-up of the patients continued until they died or end of 1992, whichever came first. When the trial began, AZT was the only antiretroviral available. Participants were randomly assigned either to receive AZT immediately or to defer treatment until they developed AIDS symptoms or had persistently low CD4 counts. The trial was blinded: the deferred group received placebo, but upon developing signs of AIDS, participants were unblinded and offered AZT if they were on placebo.

There was no statistical difference in the primary outcome between the two arms: on the immediate arm, 176 of 877 people died or progressed to AIDS versus 171 of the 872 on the deferred arm. By starting treatment before they were ill, the immediate arm participants found no more benefit from AZT than those who deferred, and they were more likely to have become resistant to the drug so that by the time they did become ill, it was no longer beneficial.

That it was disadvantageous to start early was confirmed by a long-term follow-up of the trial participants who showed statistically significant worse survival in the immediate arm. But even then matters were not straightforward, because by pooling the results of a similar trial that was conducted at about the same time as Concorde, there was no significant difference between the deferred and immediate strategies.^11^

Even though these discouraging results were based on monotherapy, and the drug resistance this approach caused, Concorde was a warning about jumping to the conclusion that early combination treatment would be beneficial.

With the growing success of combination therapy, Ho published an article in the *New England Journal of Medicine (NEJM)* in 1995 provocatively titled ‘Time to Hit HIV, Early and Hard’. He wrote that recent scientific findings and therapeutic development favoured an ‘aggressive interventional strategy early in the course of HIV-1 infection’.^12^

But the scientific findings Ho referred to were based on improved understanding of the pathogenesis of the disease. For some, this was unconvincing because it was not based on clinical data and did not consider drug side effects and long-term adherence challenges. In an article published in *The Lancet* entitled ‘Hit HIV-1 hard, but only when necessary’ by Harrington and Carpenter,^13^ the authors argued for caution and a CD4 threshold of 350 cells/µL. They stated that:

[N]o available regimen can eradicate HIV-1; all currently effective regimens may cause undesirable, sometimes life-threatening, toxic effects; and, unless regimens are strictly adhered to, multidrug resistance can develop, limiting future treatment options.

Through the 2000s, as various randomised controlled clinical trials were conducted, the when-to-start debate became increasingly nuanced. A trial showed that treating infants upon diagnosis reduced mortality by 76% and HIV progression by 75%.^14^ Two trials in adults showed that a threshold of 350 cells/µL resulted in better outcomes than 250 or 200.^15,16^ But the question of whether to treat adults irrespective of CD4 cell count, or to wait until it declined to some optimal value remained unanswered, at least in clinical trials.

## A mathematical model causes a stir

Studies of antenatal transmission of HIV as well as observational data showing that sexual transmission was more likely if the infected partner’s viral load was higher suggested that antiretroviral treatment could be used to reduce new infections.^17^ Based on these findings, Granich et al.^2^ published results of two mathematical models. They found that if a policy of universal testing coupled with the offer of immediate treatment to people who were found to be HIV-positive was introduced in South Africa, incidence and mortality because of the disease could be reduced to ‘less than one case per 1000 people per year by 2016, or within 10 years of full implementation of the strategy’. They wrote that the prevalence of HIV could be ‘less than 1% within 50 years’.

The authors included leading WHO researchers, including Kevin de Cock, the director of its HIV department. Its publication, while not responsible for starting the discussion on whether the CD4 count initiation criterion should be dispensed with and a policy of universal testing and immediate treatment should be pursued, certainly escalated the intensity of the debate. At the time of writing the article has been cited over 1600 times according to Google Scholar. This is extraordinarily high for mathematical models, the details of which most scientists, activists and policymakers are unlikely to understand, even though these were relatively simple models, which was part of their appeal.

The article’s findings were first presented ahead of World AIDS Day in 2008, and the response to it was divided. Email correspondence at the time by leaders of the Treatment Action Campaign (TAC), the leading AIDS activist organisation in South Africa, conveyed both the excitement and scepticism the article generated. The organisation’s leader, Zackie Achmat wrote, ‘This is going to overwhelm us with calls. [Our policy department] will draft a statement. The heavens are opening up’ (Achmat Z, personal communication, n.d.).

Another leader of the organisation, Mark Heywood, wrote:

I heard Kevin de Cock present this paper in Geneva … I have serious concerns about it, as does Peter Piot and most at UNAIDS! It has the potential to create a great deal of confusion, so our statement will have to be very careful. You should also be aware that in meetings to justify the paper de Cock is also claiming it has the support of activists… (Heywood M, personal communication, n.d.).

Heywood was a co-signatory on a statement by a group of ‘independent experts advising UNAIDS on HIV and human rights’ published on World AIDS Day 2008. While welcoming ‘a model that proposes the attainment of universal access to HIV treatment and HIV testing’, that ‘confirms the critical link between HIV prevention and HIV treatment’, the authors wrote the study did not ‘really address’ the problems of stigma and discrimination which could be exacerbated by potentially coercive approaches. They wrote:

To be both effective and just, programmes to scale-up HIV testing and treatment must be based on evidence and must protect the human rights of both the non-infected and the infected.

They cautioned about ‘the application of theoretical models to fictitious populations’.^18^

The publication of the Granich et al. article was accompanied by letters from accomplished researchers in various fields of HIV who criticised various aspects of the model:

- The ‘hypothesis that suppressive antiretroviral therapy can reduce HIV transmission within a sexual relationship is plausible, but unproven’, wrote Cohen et al.,^19^ scientists who within a few years would indeed prove the protective effect of treatment within a sexual relationship.
- They underestimated infectiousness in early infection and overestimated the number of partners South Africans report having, wrote Harvard demographers.^20^
- Harold Jaffe, who was at the forefront of the discovery of the AIDS epidemic, and his colleagues pointed out that the risks and benefits of treating people with a CD4 count above 350 cells/µL were unknown. They wrote, ‘Trials of therapy for patients with higher counts are yet to begin. Within the field of communicable diseases, we are aware of little precedent for the approach of “treating for the common good”.’^21^
- Ethiopian public health officials described the difficulties of implementing mass testing in a resource-limited setting.^22^

More complex models were developed in the aftermath of the Granich et al. article, though none achieved as much public discussion. Twelve models, including one of the Granich et al. ones, were described in an article by Eaton et al.^23^ The model results were compared under a set of similar assumptions about how universal testing and treatment would be carried out versus if the South African treatment guideline criteria at the time (with a CD4 initiation threshold of 350 cells/µL) were used.

The authors concluded that although the models evaluating the impact of treatment ‘vary substantially in structure, complexity, and parameter choices’, all suggested that treatment at ‘high levels of access and with high adherence’ would reduce new infections. Although there ‘was broad agreement regarding the short-term epidemiologic impact of ambitious treatment scale-up’, the models varied on their ‘longer term projections’ and ‘in the efficiency with which treatment can reduce new infections’.

One of the most sophisticated set of models aimed at determining the effect of universal testing and treatment on the epidemic was published by Hontelez et al.^24^ Explaining the motivation for their study, they wrote:

there are as many different conclusions as there are models that investigated the issue. As models are profoundly different in many aspects – structure, parameterization, and assumptions about the intervention – it is difficult to determine which factors are responsible for the differences in the model predictions. (p. 2)

The period since the publication of the Granich et al. model had also produced new evidence that the authors relied upon.

The authors developed nine structurally different models of increasing complexity, starting with one that resembled that of Granich et al. In contrast to the set of relatively simple differential equations that characterised the Granich et al. model, their most complicated models simulated people (usually referred to as agents in simulation literature) with complex algorithms for choosing sexual partners. Their results confirmed that ‘universal testing and immediate treatment at 90% coverage’ would eliminate the HIV epidemic in South Africa. But they also found that their models, which they claimed were more realistic, ‘show that elimination is likely to occur at a much later point in time than the initial model suggested’. They also found that universal testing and treatment is cost-effective, but less so than calculated by Granich et al. Most interestingly, they found that ‘the current South African … treatment policy alone could already drive HIV into elimination’.^24^

However, it is controversial whether adding complexity to models improves them. One of the authors of the Granich et al.’s article, Brian Williams, a leading figure in mathematical modelling of infectious diseases, has written a response questioning their methodology. He writes:

Hontelez et al. suggest that the current scale-up of ART at CD4 cell counts less than 350 [cells/µL] will lead to elimination of HIV in 30 years. I disagree … and believe that their more complex models rely on unwarranted and unsubstantiated assumptions.^25^

Williams’ view was that there was already sufficient evidence to make treatment universally available. He wrote:

the challenge now is to mobilize the political will and the financial support to make early treatment available to all that want it in order to save lives, save money and stop AIDS.^25^

## Treatment as prevention

Observational studies published between 2006 and 2011 showed that people with HIV on antiretroviral treatment were likely less infectious.^26,27,28,29^ But a clinical trial was needed to remove the possibility of confounding factors and estimate the magnitude of the effect.

In July 2011, the results of the HPTN 052 study were presented to a standing ovation at the meeting of the International AIDS Society in Rome. A month later the results were published. In this multinational randomised controlled trial of 1700 sero-discordant couples, the partner with HIV was randomly assigned to receive treatment immediately or to delay until 250 cells/µL. This partner also had to have a CD4 count between 350 cells/µL and 550 cells/µL at enrolment, which took place between 2007 and 2010.

Using genetic analysis, the authors found that in the immediate group, there was only one transmission to the HIV-negative partner. In the deferred group, there were 27 such transmissions, meaning that the transmission rate in the immediate group was 96% lower.^3^ To date, this remains the most beneficial HIV sexual transmission prevention effect found in any randomised controlled clinical trial.

The study also found that there were clinical benefits for patients who started earlier, but as the initiation threshold was 250 cells/µL, a point already known to be lower than optimal (although the most convincing clinical trial showing this had not yet completed at the time HPTN 052 enrolled), it did not resolve the when-to-start debate, at least not from the perspective of the individual patient. However, it did result in the WHO publishing guidelines that recommended immediate treatment – for the purpose of prevention – for HIV-positive people with HIV-negative sexual partners.^30^

The debate on when-to-start swung noticeably towards earlier treatment after the publication of HPTN 052. Here is some of the discussion that followed.

Joseph Sonnabend, a physician, wrote a blog expressing caution against immediate treatment of anyone who tested positive and had a CD4 count above 350 cells/µL:

The recent demonstration that antiretroviral treatment can prevent transmission of HIV among sero-discordant heterosexual couples is great news. However, when the person offered treatment has not yet been shown to personally benefit from it, an ethical issue needs to be addressed.^31^

In an interview, the study’s principal investigator, Myron Cohen, stated his support for the earlier treatment recommendations made to the US guidelines following HPTN 052. ‘That’s a pretty big change’, he said, ‘and it respects the accrued benefits, which are very, very strong’.^32^

In a critical response to Cohen, AIDS activist Simon Collins^33^ wrote:

a radical public health approach to HIV care is presented as self evident, while neglecting to discuss the lack of important data or presence of contradictory evidence. This is a serious omission in an historical context of guideline recommendations that have been wrong on this question more often than they have been right.

He further wrote:

Even with the best intentions, guidelines produced by experts, can be wrong. The limited evidence and lack of randomised data, restricts the ability to know the risks as well as the benefits.^33^

Given the state of uncertainty about the optimal initiation threshold and that many sexually active people would want to start treatment to reduce their infectiousness, Collins and Geffen wrote:

the decision of when to start must be taken by the HIV-positive person in consultation with their health worker based on accurate information. That choice will vary depending on a person’s individual health, their reason to want to treat and the resources of the health-care facility.^9^

## Observational data

In April 2009, two large studies were published that had a considerable impact on the when-to-start debate. Both used observational data to calculate the effect on mortality of different CD4 count initiation thresholds.^34,35^

Kitahata et al.^34^ studied over 17 000 Canadian and US patients. They found a substantial increase in the risk of death for people who deferred treatment below a CD4 count of 500 cells/µL. Those who deferred to below 350 cells/µL had the highest risk of death. However, the study used novel methods that introduced bias in favour of earlier treatment. The authors were criticised for this in several subsequent letters to the editor. They responded that even taking these concerns into account, their data still supported earlier treatment.

An accompanying editorial pointed out that the strengths of this study:

included its relatively large size, the use of advanced statistical methods that attempted to analyze the data in a fashion similar to that of a randomized trial, and the use of survival … as the end point.^36^

Nevertheless:

the results of the … study cannot be considered definitive evidence that everyone with HIV should start receiving antiretroviral therapy. This was not a randomized trial, and the patients who chose to begin therapy early might have differed in other important ways from those who chose to defer therapy – ways that improved survival but were not measured.^36^

The editorial concluded that if five years previously an asymptomatic patient with HIV with a CD4 cell count above 500 cells/µL wished to start treatment, most experienced clinicians ‘could have made an excellent case’ for deferring treatment.

Today, if a similar patient were eager to start, we should be ready and willing to prescribe therapy – with ongoing careful monitoring of toxic effects that could arise during decades of treatment.^36^

But the UK funded when-to-start consortium,^35^ which looked at over 21 000 patient records, had less convincing results with less controversial methods. The authors found that deferring therapy to a CD4 count of 250–350 cells/µL was associated with higher rates of a composite endpoint of AIDS or death than deferring to 351–450. However, when mortality alone was considered, there was no statistical significance. And at stepwise comparisons of higher CD4 count ranges, they could find no significant difference in the primary outcome. The authors noted, ‘The evolution of guidelines has been compared to the swings of a pendulum’. They motivated for a 350 cells/µL threshold.

Subsequently, UK and US guidelines diverged, with the latter taking steps in subsequent editions that promoted earlier treatment.

Jain and Deeks^37^ summarised the situation at the time:

Although the debate regarding when to start antiretroviral therapy has been present for over two decades, consensus on this question has been hard to achieve. This lack of clarity continues in the current era, with major guidelines recommending very different treatment strategies. All agree, however, that the pendulum has swung back in favor of more aggressive approaches to therapy. The philosophy of delaying potentially toxic medications as long as possible has increasingly shifted toward a philosophy of initiating therapy as soon as possible.

This shift was evident when UNAIDS published its 90–90–90 strategy in October 2014.^38^ The second of the three 90s referred to having 90% of people diagnosed HIV-positive on sustained antiretroviral treatment by 2020 – a target that amounts to an endorsement of test and treat. But this was at odds with the WHO’s treatment guidelines, which at the time only recommended treatment initiation at CD4 counts of 500 cells/µL or below.^39^

In his budget vote speech in July 2014, shortly after returning from the 20th International AIDS Conference in Melbourne Australia, South Africa’s Minister of Health endorsed the 90–90–90 targets and treatment irrespective of CD4 count. While he endorsed test and treat, he only went as far as announcing that the treatment initiation threshold would be raised from 350 cells/µL to 500 cells/µL.^40^ In response, the TAC’s policy director criticised Motsoaledi for recommending earlier treatment initiation without consulting activists.^41^

## Strategic Timing of Antiretroviral Treatment (START)

Members of the trial’s community advisory board wrote that the Strategic Timing of Antiretroviral Treatment (START) trial:

is a study that has been driven by community demand that the optimal clinical initiation threshold for [antiretroviral treatment] be determined by clinical trial evidence rather than expert opinion informed primarily by observational data.^42^

START was conceived in the mid-2000s to resolve definitively the question when it would be best to start treatment from the perspective of a patient with HIV. The trial was randomised but open-label because a placebo arm would have created insurmountable practical and ethical problems.^4^

Nearly 4700 people enrolled in the trial at 215 sites in 35 countries between April 2009 and December 2013. To participate, patients had to be antiretroviral treatment naive, and have a CD4 cell count greater than 500 cells/µL. Participants were randomised either to begin treatment immediately or to wait until their CD4 counts dropped to 350, or treatment was clinically indicated. The primary endpoint was a composite of any serious AIDS- or non-AIDS-related event, or death.^43^

Support for the trial was not universal. Franco and Saag^44^ wrote that the balance of data strongly supported starting treatment in nearly everyone regardless of CD4 count. They cited the availability of better drugs that were now available, the current understanding of HIV biology and pathogenesis and evidence from observational data. They conceded that a small group of people who ‘have undetectable virus in the absence of antiretroviral therapy’ might be exceptions. But for:

everyone else, to wait on randomized clinical trial data could well be doing harm. The time spent waiting is time that the patients cannot get back and the long-term damage associated with waiting could well be irreversible.^44^

By the time they wrote this, however, START was well underway.

Contrast this with a view expressed by some of the main researchers involved in the publication of the observational data, after the US guidelines changed. Phillips et al.^45^ wrote ‘We are concerned that some may interpret the new recommendations as implying that the deferral group of this trial [START] is no longer ethical. Such an interpretation would endanger the future of the trial in the USA’. After explaining the problems with the observational data, they concluded:

We therefore do not believe that there is convincing evidence to conclude that deferral of initiation of ART to a CD4 count of 350 causes net harm, particularly in terms of mortality, compared with starting at any higher level. We strongly support continued enrolment into START. Large randomised studies represent the only means of eventually obtaining the definitive result we need to properly inform future patient care.

The trial was only expected to produce results in late 2016 or early 2017. But in May 2015, the trial’s independent data and safety monitoring board informed the main sponsor that the question had been answered. It recommended that the findings be ‘immediately disseminated’. The primary endpoint occurred in 42 people in the immediate arm versus 96 in the deferred one, meaning the risk of serious illness or death was less than half in the immediate one (though for an HIV cohort, patient outcomes were good in both arms). Even at high CD4 counts, treatment reduced the risk of AIDS illnesses.^43^

At about the same time as START, a smaller trial (2076 participants) called TEMPRANO was run in Côte d’Ivoire. Primarily concerned with the effect of earlier treatment in an area with high tuberculosis, it had a similar primary endpoint to START. Its findings, which also showed the benefit of immediate treatment, were made public shortly before START’s were. However, the details of the study were presented at the International AIDS Conference in Vancouver on the same day as START’s and both studies were published in the same journal on the same day.^46^

The last outstanding piece of evidence in the when-to-start controversy had now been answered.

## Discussion

There were two main points of contention in the when-to-start debate.

First, there was disagreement over what constituted sufficient evidence that early treatment was beneficial. There was an implicit hierarchy of evidence, with biological plausibility and mathematical models constituting the lowest evidence, followed by observational data. For many, this was sufficient to make the case for immediate treatment.

But those who considered observational data to be too uncertain and prone to confounding demanded a randomised clinical trial. They were concerned by the additional adherence demanded of patients who started treatment early in their HIV infection, the side effects primarily associated with earlier generations of antiretroviral drugs, the unfortunate experience of Concorde and the fluctuations of guideline recommendations in the absence of compelling evidence. There was also concern that the public health concern of the reduced infectiousness of people with HIV was taking precedence over the uncertainty about the benefit of immediate treatment for the health of individuals with HIV. The publication of the START results resolved these points of contention.

## Conclusion

There are no accepted criteria for resolving scientific debates with policy repercussions when the evidence is still being gathered. In contrast to the destructive and irrational debate on the cause of AIDS that took place in South Africa in the 2000s, the protagonists on both sides of the when-to-start debate included leading experts in HIV science who could draw on substantial evidence to make their arguments. In the case of AIDS denialism, one side of the debate shunned the immense body of evidence, preferring conspiracy theories instead, whereas on the question of when-to-start, the science truly was in dispute (see^1,47,48,49^ for further discussion on this). Now that all the data, including the gold standard of medical science – a randomised controlled trial – support immediate treatment, continuing to advocate for delayed treatment on medical grounds would be irrational.
